# Patient age is related to decision-making, treatment selection, and perceived quality of life in breast cancer survivors

**DOI:** 10.1186/1477-7819-12-230

**Published:** 2014-07-22

**Authors:** Terence T Sio, Kenneth Chang, Ritujith Jayakrishnan, Difu Wu, Mary Politi, Dominique Malacarne, James Saletnik, Maureen Chung

**Affiliations:** 1 Department of Surgery, Warren Alpert Medical School of Brown University, Providence, RI, USA; 2 Department of Radiation Oncology, Mayo Clinic, 200 First St SW, Rochester, MN 55905, USA; 3 Department of Surgery, Washington University, St. Louis, MO, USA; 4 Taubman Center for Public Policy and American Institutions, Brown University, Providence, RI, USA; 5 Margie and Robert E. Petersen Breast Cancer Research Program, John Wayne Cancer Institute at Saint John’s Health Center, Santa Monica, CA, USA

**Keywords:** Breast cancer, Oncology, Age, Decision-making, Quality of life

## Abstract

**Background:**

Patients with breast cancer must choose among a variety of treatment options when first diagnosed. Patient age, independent of extent of disease, is also related to quality of life. This study examined the impact of patient age on treatment selected, factors influencing this selection, and perceived quality of life.

**Methods:**

A 62-question survey evaluating breast cancer treatment and quality of life was mailed to breast cancer survivors. Responses were stratified by age (<50, 50-65, >65 years) and extent of disease.

**Results:**

Of the 1,131 surveys mailed, 402 were included for analysis. There were 104, 179, and 119 women aged <50, 50-65, and >65 years, respectively. The median patient age was 58 years, and the average interval from diagnosis to survey participation was 31.5 months.

**Conclusions:**

Young women were more likely to have undergone aggressive therapies and had better physical functioning than old women. Old patients reported good quality of life and body image. Clinicians should consider patient age when discussing breast cancer treatment options.

## Background

Early detection and improved multimodality therapy have improved breast cancer survival [1]. When first diagnosed, patients with breast cancer must make decisions about the type of surgery and adjuvant therapy, each with its own risks and benefits. The decision-making process is complex, and is influenced by multiple factors including patient age, co-morbidities, ethnicity, education, and availability of visual and written hand-outs. Patient age is so important that it has been proposed as a determinant of educational and counseling strategies [2,3].

Age at diagnosis may also influence patient behavior and outcome. Older women prefer to take a more passive role in decision-making, relying heavily on their doctors’ recommendations [3], whereas younger women are more actively involved in treatment decisions, seeking information from multiple sources such as the Internet, friends, family members, and support groups. It has been found that patients provided with a greater amount of medical information were associated with less depression and improved quality of life [4]. In the same study, patients who were satisfied with their participation in the decision-making process were also found to be less depressed. Younger women may desire treatments that they perceive increase life expectancy, while older women may prefer treatments that maximize quality of life [5,6].

The current study was a retrospective analysis of treatment selection and perceived quality of life in a cohort of breast cancer survivors. We hypothesized that age at diagnosis of breast cancer significantly influenced patient selection of type and duration of therapy, independent of extent of disease, and that it was directly related to perceived quality of life after treatment. A cross-sectional and correlational study design was used.

## Methods

The Comprehensive Cancer Center of Rhode Island Hospital is a tertiary cancer center that treats approximately one-third of all patients diagnosed with breast cancer in Rhode Island. All breast cancer cases are presented prospectively at the multidisciplinary breast tumor board and recorded in the Rhode Island Hospital Breast Cancer database. Patients with breast cancer diagnosed from January 2004 to June 2007 were identified from this database. Female patients with invasive breast cancer or ductal carcinoma *in situ* without known recurrent disease were included. Male patients were excluded.

A survey evaluating breast cancer treatment and quality of life was mailed to patients with breast cancer on 15 July 2008 (Additional file 1). Patients were asked to complete the survey and return it anonymously in a pre-stamped addressed envelope. A raffle was used to increase survey participation, but the raffle form was returned in a separate pre-stamped addressed envelope to protect the anonymity of participants. Both English and Spanish versions of the survey were available. The English version of the survey was mailed initially with information on how to obtain the Spanish version included at the top of the survey. This study was approved by the Rhode Island Hospital Institutional Review Board.

The European Organization for Research and Treatment of Cancer (EORTC) QLQ-C30 [7] and BR23 [8] questionnaires were utilized in the survey. The QLQ-C30 module includes global health, functional, and symptom subscales which are robust survey tools for psychometric evaluation and reproducible across population [7]. The BR23 module evaluates functional and treatment-related symptoms specific to breast cancer, including body image questions. Additionally, the survey included 56 independently developed questions (supplementary section) related to cancer characteristics (4), demographics (10), extent of disease (2), use and type of surgery (6), radiotherapy (6), chemotherapy (7), anti-hormones (5), support systems (10), and perception of therapies (6). Decisions about treatment were collected by asking patients to identify the surgery their clinician recommended, the reason for the recommendation, if known, and the surgery chosen. The survey also asked participants to identify whether radiotherapy, anti-hormone medication, or chemotherapy was recommended, and their final treatment selected. Patients were provided a list of personal, social, and professional support structures, and were asked to select the three which most influenced their treatment decision. Eight questions assessed how patients felt about their treatment choices and perceived quality of life. The Decision Regret Scale, a validated five-item scale for measuring regret after treatment choices, was used to assess patient satisfaction with treatment selected [9]. Perceived quality of life was measured by the EORTC QLQ-C30 [7] and the QLQ-BR23 [8], and included questions about fear of recurrence and death.

We used the average age at onset of menopause (50 years) and the qualifying age for Medicare benefits (65 years) to divide patients into three age groups: young (<50 years), middle-aged (50-65 years), and old (>65 years). Extent of disease (local represented disease confined to the breast and regional indicated disease that had spread to regional lymph nodes) and treatment differences were analyzed for each age group. Correlations between quality of life and social support, decision regret, education, and income were determined. Raw scores were calculated according to the Likert scale of individual surveyed items. Linear transformation was used to convert raw scores of global health status and functional scales to a scale of 0 to 100, with 100 being *highest level* of function and quality of life. Symptom scales (examples: lymphedema, hair loss) were scored from 0-100, with 100 being *most symptomatic*. Future perspective of patients was scored from 0-100, with 100 being *most optimistic* about their health and prognosis. The Decision Regret Scale ranged from 1-5, with 5 being *strongly agree*.

### Statistical analysis

A chi-square test was used to compare categorical results and variables across age groups. Fisher’s exact test was used to calculate *P*-values when 20% of the cells had values below 5. For continuous variables, analysis of variance (ANOVA) or Kruskal-Wallis tests for three or more groups (when the data were skewed) were conducted. Logistic regression models were used for continuous predictor variables with dichotomous outcome variables. The statistical significance was set at *P* < 0.05. Data were analyzed using SAS version 9.2 software.

## Results

### Demographics

Of 1,131 surveys mailed, 525 (46%) were returned and 402 were used for analysis (111 were excluded from analysis because they did not meet inclusion criteria due to factors such as unknown year of diagnosis or incomplete surveys; 12 additional patients were excluded from the analysis due to systemic disease at initial diagnosis). No respondents requested the survey in Spanish. The median patient age was 58 years (range 29 to 97), and 96.3% of the surveyed population was Caucasian. The average interval between diagnosis and survey completion was 31.5 months and did not vary by age group (*P* = 0.25). Stratified by age, patient characteristics and extent of disease are summarized in Table 1. There were 104 (26%) young women with an age less than 50 years old, 179 (44%) middle-aged women 50 to 65 years old, and 119 (30%) old women greater than 65 years old. Young women were more likely to be diagnosed with regional disease than old women. Old patients were more likely to be single/divorced or widowed and less formally educated. Young patients were more likely to experience financial difficulties due to breast cancer. Although they experience equal rates of disability, younger women were more likely to be disabled from breast cancer, while older women were more likely to be disabled from another health condition. Support group participation was underutilized across all age groups.

**Table 1:** Demographics and cancer characteristics of study cohort, stratified by age

	**Age < 50**	**Age 50-65**	**Age >65**	***P*** *
	**n (%)**	**n (%)**	**n (%)**	
Extent of disease				
Confined to the breast (local)	63 (63.0)	127 (79.9)	81 (79.4)	0.011
Lymph node involvement (regional)	37 (37.0)	32 (20.1)	21 (20.6)	
Caucasian	99 (98.0)	172 (96.1)	113 (95.0)	0.870
Marital status				
Single/divorced/widowed	21 (20.2)	48 (26.8)	56 (47.5)	<0.001
Married/partnered	83 (79.8)	131 (73.2)	62 (52.5)	
Education beyond high school	82 (78.8)	120 (67.0)	53 (44.5)	<0.001
Support group participation	11 (10.7)	18 (10.2)	10 (8.5)	0.912
Has one or more children	83 (79.8)	142 (79.3)	106 (89.1)	0.093
Financial difficulties due to breast cancer				
Not at all/a little	86 (84.3)	159 (89.8)	110 (94.0)	0.063
Quite a bit/very much	16 (15.7)	18 (10.2)	7 (6.0)	
Has disability	13 (12.6)	18 (10.2)	14 (11.8)	0.993
From breast cancer	6 (5.8)	1 (0.6)	0 (0.0)	0.010
From breast cancer and other condition	4 (3.9)	8 (4.5)	4 (3.4)	
Unrelated to breast cancer	2 (1.9)	7 (4.0)	9 (7.6)	

*P values apply to comparison across all three age groups.

### Determinants of treatment decision

The surgical options offered by the physician differed significantly between each age group (*P* < 0.0001), with lumpectomy being more recommended than mastectomy. Furthermore, physicians were less likely to recommend chemotherapy to older patients (*P* < 0.0001). However, there was no observable difference between age groups for a physician to recommend radiation therapy (*P* = 0.24) or anti-hormone therapy (*P* = 0.06). When stratified by age and disease extent, young women were more likely than old patients to have undergone aggressive therapy (Table 2). Young and middle-aged women were more likely to have chosen mastectomy than their older counterparts (*P* < 0.0002 [odds ratio (OR) = 4.68] and *P* < 0.011 [OR = 2.46], respectively). Medical therapies were utilized to a greater extent in the young age groups (Table 2). For women with localized disease, young women were more likely to have received chemotherapy than middle-aged or old women (*P* = 0.027, OR = 1.98). Similar results were obtained for women with regional disease (*P* = 0.014, OR = 9.59).

**Table 2:** Extent of disease and cancer therapy stratified by patient age

**Total number of patients in each age group (N)**	**Age <50**	**Age 50-65**	**Age >65**	***P***^**1**^
	**n (%)**^**2**^	**n (%)**^**2**^	**n (%)**^**2**^	
	**N = 104**	**N = 179**	**N = 119**	
Surgery for disease localized to the breast				0.002
Lumpectomy	41 (39.4)	91 (50.8)	68 (57.1)	
Mastectomy	20 (19.2)	30 (16.8)	6 (5.0)	
Surgery for regional disease				0.158
Lumpectomy	16 (15.4)	20 (11.2)	12 (10.1)	
Mastectomy	20 (19.2)	11 (6.1)	6 (5.0)	
Prophylactic mastectomy	12 (11.5)	19 (10.6)	4 (3.4)	0.046
Systemic chemotherapy				
For disease localized to the breast	24 (23.1)	31 (17.3)	10 (8.4)	0.027
For regional disease	34 (32.7)	25 (14.0)	14 (11.7)	0.014
Radiation therapy				
For disease localized to the breast	37 (35.6)	80 (44.7)	52 (43.7)	0.125
For regional disease	31 (29.8)	25 (14.0)	17 (14.3)	0.921
Anti-hormone therapy	72 (69.2)	107 (59.8)	52 (43.7)	0.089

^1^*P* values apply to comparison across all three age groups.

^2^The percentages in the table were generated by dividing the number of patients who fell under the corresponding criteria by the total number of patients in the age group as listed; due to missing values, some percentages may not add up to unity.

Age and extent of disease influenced surgical therapy. Independent of age, patients with regional breast cancer were more likely to have had a mastectomy. Young patients with localized breast cancer were also more likely to have had a mastectomy than their older counterparts. Patient age also influenced systemic adjuvant therapy. Young patients with breast cancer were more likely to have received cytotoxic chemotherapy and/or anti-hormonal therapy. No difference in receipt of breast radiotherapy was observed based on patient age. The odds ratio of choosing mastectomy for young women (<50) and older women >65 was 4.186 (confidence interval 1.996 to 8.781, *P* < 0.02). The odds ratio of choosing mastectomy for women 50-65 compared to those >65 was 2.526 (confidence interval 1.238 to 5.152, *P* < 0.01).

The three most important factors that influenced treatment choice were surgeon’s recommendation (83.1%), desire for longevity (72.6%), and family or significant others (59.1%). Young patients (87.9%) were more likely to want to live as long as possible compared to middle-aged (71.1%) and old women (67.7%) (*P* = 0.0016). Factors that influenced choice of treatment but had no statistically significant difference by age groups were self-image (*P* = 0.74), treatment side effects (*P* = 0.74), other factors (friends, cost, religion) (*P* = 0.61), and length of therapy (*P* = 0.13). Overall, 90.7% of patients were “satisfied” or “very satisfied” with the treatment information provided by their physicians. The majority of patients reported little to no regret for the treatments that they eventually chose: 89%, 89%, and 86% of patients reported no or mild regret (scores, 0-25) among the young, middle-aged, and old women, respectively (*P* = 0.74).

### Quality of life

**Figure 1:**
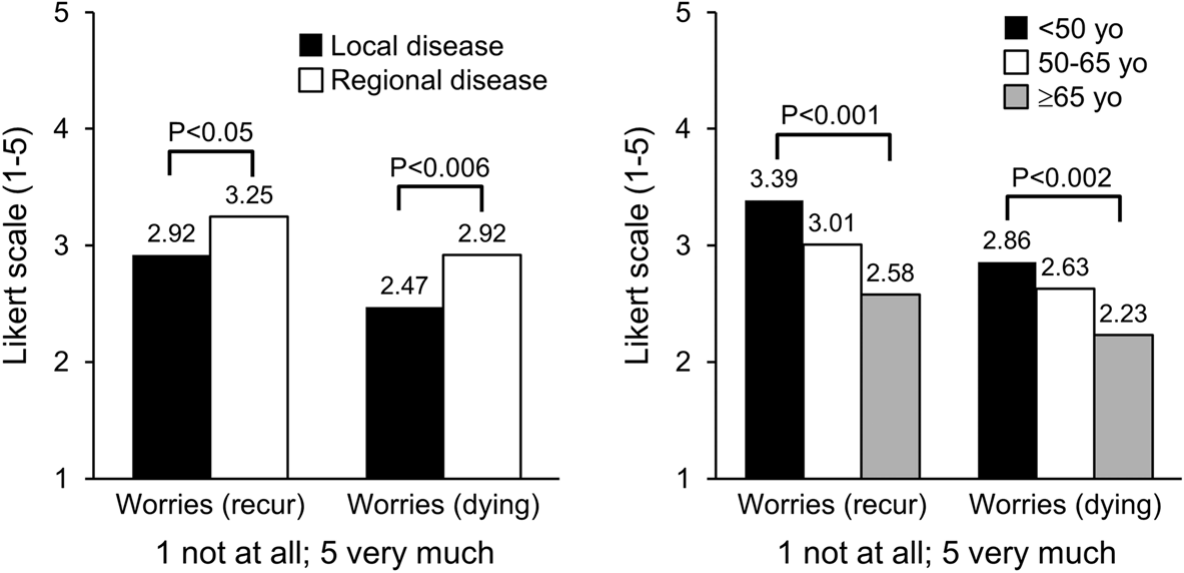
Worries about recurrence and dying in patients with breast cancer.

In general, old women reported poorer general health and functioning than middle-aged and young women (Table 3). Most respondents had good quality of life with minimal treatment-related symptoms. Across age groups, there was no difference in morbidity associated with side effects (for example, alopecia and lymphedema) from cancer therapies. Nevertheless, young women had poor body image compared to old women. Young women were also more concerned about recurrence and dying compared to middle-aged and old women. Women with regional disease worried more about recurrence and death, compared to women with disease confined to the breast (Figure 1). After controlling for extent of disease, age was significantly related to physical functioning (*P* < 0.001) and future perspective (*P* < 0.001), but was not related to global health status (*P* > 0.06) or breast symptoms (*P* > 0.06). Extent of disease was significantly related to global health status (*F* = 4.2, *P* < 0.04, data not tabulated). Women with regional disease reported poor quality of life compared with those with local disease (79.1 versus 83.7). Compared to those with local disease, they were also more affected in physical (80.5 versus 86.6, *F* = 7.05, *P* < 0.008) and role functions (83.3 versus 89.3, *F* = 4.5, *P* < 0.04). These data suggested that extent of disease may independently affect quality of life.

**Table 3:** Quality-of-life characteristics by age

	**Age <50**	**Age 50-65**	**Age >65**	***P*** *
	**Mean (SD)**	**Mean (SD)**	**Mean (SD)**	
Global health status (2)	83.4 (19.8)	84.1 (18.3)	80.5 (17.5)	0.057
Physical functioning (5)	89.7 (16.5)	86.7 (17.8)	78.9 (21.3)	<0.001
Role functioning (2)	85.9 (27.4)	86.7 (24.4)	90.3 (18.0)	0.839
Body image (4)	69.7 (29.6)	82.8 (22.4)	90.4 (18.2)	<0.001
Sexual functioning (2)	38.8 (24.5)	27.6 (26.2)	16.2 (22.6)	<0.001
Sexual enjoyment (1)	56.7 (33.0)	45.6 (34.6)	31.5 (33.0)	<0.0002
Future perspective (1)	46.7 (34.9)	55.2 (32.0)	70.6 (28.3)	<0.001
Side effects from chemotherapy (8)#	17.3 (16.8)	16.0 (16.3)	13.3 (13.5)	0.107
Breast symptoms (4)#	15.7 (18.5)	15.7 (19.3)	10.7 (11.8)	0.210
Arm symptoms (3)#	17.0 (26.2)	13.1 (21.4)	8.4 (13.9)	0.135
Distress from loss of hair (3)#	9.8 (25.0)	17.2 (31.7)	19.4 (31.1)	0.092
QLQ-BR23 total (23)	75.1 (14.3)	78.1 (14.5)	82.3 (10.8)	<0.001

Parentheses indicate number of questions asked in each subscale.

#Symptom scales applied. *P values apply to comparison among all three age groups. SD represents standard deviation. Nonparametric Kruskal-Wallis tests were used for comparison.

The survey results of the EORTC QLQ-C30 and BR23 subscales are shown here, stratified by age group. Overall, older patients reported a better quality of life after breast cancer therapy. Although younger patients with breast cancer reported better physical functioning, older patients had a better body image. All patients reported equivalent treatment-related side effects.

Menopausal status is a potential confounder in health-related quality-of-life analysis, as it is physiologically related to age and sexual health. Menopause has been shown to significantly diminish the quality of life of women [10]. After controlling for menopausal status, age was significantly related to sexual functioning (*P* < 0.0001) and enjoyment (*P* < 0.0009), with old women more negatively affected. Menopausal status was also related to body image (*F* = 16.45, *P* < 0.0001 favoring older) and future perspectives (*F* = 5.54, *P* < 0.02 favoring older). However, there was no relationship between menopausal status and total EORTC QLQ-BR23 score (*F* = 1.56, *P* = 0.21), arm symptoms (*F* = 0.01, *P* = 0.92), breast symptoms (*F* = 0.90, *P* = 0.34), chemotherapy side effects (*F* = 0.04, *P* = 0.84), and alopecia-associated distress (*F* = 0.27, *P* = 0.60). Old women were more likely to have received or were recommended anti-hormonal medication. After controlling for anti-hormonal therapy, age was significantly related to total BR23 score (*F* = 6.61, *P* < 0.002), body image (*F* = 16.79, *P* < 0.0001), and sexual function (*F* = 13.82, *P* < 0.0001). Age was significantly related to body image (*P* < 0.0001) after controlling for mastectomy use.

## Discussion

Breast cancer therapy consists of multimodality treatment ranging from breast conservation to mastectomy, whole or partial radiotherapy of varying duration, selective use of anti-hormone medication, and chemotherapy regimens that differ in duration and morbidity. Our data supported the original hypothesis that age is related to treatment choice; young women were more likely to choose aggressive therapies, even when diagnosed with localized disease. This result is consistent with the observation that young women may select more aggressive treatments in order to maximize their survival [11,12]. By contrast, Bleicher and colleagues [3] reported no difference in mastectomy choice by age, and a study from the University Hospital of Wales reported that older patients were more likely to choose mastectomy as a treatment option [13].

Increased awareness of family history may increase the likelihood of young patients pursuing mastectomy. Although these variables were not included in our survey, it has been reported that women with a family history of breast cancer were more likely to choose mastectomy as their breast cancer treatment option [14]. Women with a family history of breast cancer are also more likely to undergo prophylactic mastectomy [15]. This may partially explain why young women in our study were more likely to undergo more invasive surgery, including prophylactic mastectomy, because the risk of recurrence and desire for longevity are significant patient concerns.

The need for maximal survival may influence surgical treatment choice [16]. Patients with breast cancer may opt for mastectomy, as they perceive that this procedure increases cancer survival. Although local recurrence decreases with mastectomy, breast-conserving surgery is no less efficacious than mastectomy with regard to long-term survival [16]. In general, a higher education level was associated with opting for breast conservation, which may be due to a better understanding of risk recurrence (Surveillance, Epidemiology, and End Results (SEER) Program, Fast Stats-Breast Cancer). However, young patients in our study were better educated but less likely to choose breast-conserving surgery. The increased use of mastectomy by young women may be a reflection of feeling comfortable with invasive surgery due to having a greater social support network of family and friends. In one prior study, elderly breast cancer survivors reported having less social support network mechanisms than young women [17]. In the population studied in this report, an increase in support group utilization across all age groups may present a future research direction.

In addition to choosing less invasive surgery, older women were also less likely to receive chemotherapy. These women were less likely to use anti-hormone medication despite their physicians’ recommendations. Furthermore, older women tend to have poorer general health than young women, and are more likely to have medical co-morbidities [17]. This may, in part, explain why more older women chose not to receive chemotherapy or anti-hormones as part of their breast cancer treatment.

In our study, physician recommendation was of primary significance in influencing the patient’s selection of treatment. Physicians may be less likely to recommend medical interventions to older patients because of potential complications and increased morbidity [18]. The authority for decisions regarding treatment may not be equally shared between physicians and patients. Physicians may also underestimate the influence of the words they choose to describe extent of disease and potential treatments options to patients [19].

Overall, patients in this study reported being satisfied with information regarding treatment options. In addition, 83% of survey participants stated that their surgeon’s recommendation was a major influence on treatment selection options. A previous study which reviewed factors influencing surgery choice in women also showed similar results [20]. Another study showed that 95% of study participants were satisfied with time spent discussing treatment options with their surgeons, underscoring a surgeon’s influence on the type of breast cancer surgery selected [21]. The congruency between the patients’ and physicians’ opinions has been associated with treatment satisfaction, which in turn correlates with higher levels of quality of life [22]. Similarly, another group found that the more the patient was involved in decision-making, the better the quality of life that she seemed to achieve by association [23,24]. These results reiterate the importance of physician’s participation in empowering and educating patients with breast cancer regarding their treatment options.

Most breast cancer survivors reported preserved quality of life and function in this study. Breast cancer survivors’ quality of life improves with time and is comparable to the general population five years after treatment [25]. Over longer time periods, most breast cancer survivors have functional scales comparable to those of the general population [26,27]. Interestingly, patients in this study had a good quality of life after only an average of 31 months after diagnosis, suggesting that a return to function occurs much earlier than previously reported. There was no difference in side effects between the different age groups but, after controlling for extent of disease, age was significantly related to arm symptoms. In other studies, quality of life was lower in breast cancer survivors who experienced arm symptoms, with or without lymphedema [28]. This might be expected in younger women who undergo more aggressive surgery and/or are more physically active and therefore more likely to notice functional limitations.

Poorer body image in young breast cancer survivors may reflect increased selection of mastectomy as a treatment option [29]. Concerns for appearance have led younger patients to opt for lumpectomy more frequently compared to their older counterparts [30]. It has also been reported that young patients significantly experienced more worries regarding their finances and future health, and had poorer emotional and social functioning compared to the older generation [31]. Although socioeconomic status had no correlation with treatment decisions or self-perceived health-related quality of life, factors such as annual household income, disability status, and insurance coverage options may be barriers to health care access and consequently outcome in some communities [32].

There are several limitations to this study. The conclusions of the study are based solely on patient responses and may reflect a recall bias. Our survey tools were based on the EORTC QLQ-C30 and QLQ-BR23 questionnaires, which do not evaluate social and spiritual quality of life [33]. Although the survey was generated in an academic urban center, the vast majority of respondents were Caucasian with private health insurance. Thus, future studies which incorporate minority populations will be desirable, since cultural belief may represent a major factor in medical decision-making.

The survey response sample data set in our study was self-selected. Although the survey was mailed to all patients who were treated between 2004 and 2008, those who responded to the survey might have had better quality of life and health outcomes than those who chose not to respond. Breast cancer survivors may report fewer symptoms and better quality of life if they have not had a recurrence or a therapy-related complication [34]. This may have introduced a non-random sampling bias into the study results. Characteristics of survey participants and non-participants could not be compared because of the anonymous nature of the study.

Perceived quality of life may differ based on ethnicity [35] or on whether patients had private health care insurance. The initial survey was written in an English language format, which may have decreased the survey response rate of patients who speak English as a second language or not at all. A Spanish language survey format was also available to patients but only upon request.

## Conclusions

This study demonstrated that patient age plays a major role in how patients with breast cancer choose their treatments and its impact on perceived quality of life. Additionally, age is significantly related to psychosocial well-being in breast cancer survivors. Breast cancer survivors younger than 50 years were more likely to choose aggressive therapies compared to their older counterparts, while older women wanted the least disruptive treatments, choosing treatments that maximized quality of life. Age was related to treatment decision-making and quality of life in this cohort of breast cancer survivors, which validated the findings of previous similar studies. Regardless of age, most breast cancer survivors reported little to no regret regarding their treatment choices, with similar factors influencing treatment selection across all ages. A patient’s age should be considered when counseling patients about treatment options and discussing long-term quality-of-life goals in order to best individualize breast cancer care.

## Abbreviations

EORTC: European Organization for Research and Treatment of Cancer; OR: odds ratio; QLQ: Quality of life questionnaire; SEER: Surveillance, Epidemiology, and End Results.

## Competing interests

The authors declare that they have no competing interests.

## Authors’ contributions

TTS, DW, MP, and MC designed the study, collected the data, and drafted the manuscript. TTS, DW, KC, RJ, and DM performed the literature review. TTS, KC, RJ, MP, and JS performed the statistical analyses. KC, RJ, DM, and JS also participated in the drafting of the manuscript. All authors read and approved the final manuscript.

## Supplementary Material

Additional file 1 A survey on quality of life for patients with breast cancer. Click here for file
