# Supplementary material for: Patient age is related to decision-making, treatment selection, and perceived quality of life in breast cancer survivors
Source: World J Surg Oncol. 2014 Jul 22;12:230. doi: 10.1186/1477-7819-12-230 (PMC4113127; doi:10.1186/1477-7819-12-230)
Supplement: Additional file 1 — A survey on quality of life for patients with breast cancer. [file 1477-7819-12-230-S1.doc]

**Additional material**

**A Survey on Quality of Life**

**Esta Encuesta también esta disponible en Español, llamando al 444-4850**

**Dear Patient**: You have been selected to participate in this survey on quality of life in breast cancer survivors. We are interested in how you feel about your condition, the choices you had to make, and who or what may have helped you make your choices. We would greatly appreciate your help. This survey will take about 15 minutes. Please circle the answer that best shows how you feel. This survey is confidential. Please do NOT put down your address or your name anywhere on this survey. If you do not feel comfortable answering any of the questions in this survey, please feel free to skip them. Thank you.

**Please tell us about your breast cancer treatments.**

1. When were you first diagnosed with breast cancer?

Year:

Month:

(year/month, e.g. 2001/December)

2. What stage was your cancer diagnosis?

Stage 0 Stage I Stage II Stage III Stage IV Don’t know

3. When you were first diagnosed, had your breast cancer: spread to any lymph nodes under the arm? No Yes

…spread to the other breast? No Yes …spread to other organs of the body? No Yes

4. How well do you feel your treatment options were explained by your doctor?

Not at all A little bit Somewhat Quite a bit Very much

5. If you are receiving treatments today, what treatments are you receiving?

Radiation Chemotherapy Hormone therapy Other:

6. Are you receiving any hormonal therapy such as tamoxifen (Nolvadex®, Istubal®, or Valodex®), or an aromatase inhibitor (AI) such as Exemestane (Aromasin®) or Anastrazole (Arimidex®)? No Yes

7. What surgical treatment options were offered for the breast?

Total mastectomy Partial mastectomy (lumpectomy)

8. What breast surgery did your doctor recommend the most?

Total mastectomy Partial mastectomy (lumpectomy) Initially partial but eventually needed total mastectomy

9. Did you seek a second opinion? No Yes

10. If yes, what surgery did the second doctor recommend? Total mastectomy Partial mastectomy (lumpectomy)

11. What breast surgery did you choose? Total mastectomy Partial mastectomy (lumpectomy)

Initially partial but eventually needed total mastectomy Did not undergo breast surgery

12. Did you have surgery for the other breast (prophylactic mastectomy), even though the cancer had not spread to the other breast? No Yes

13. Of the following, please CIRCLE three items that have most influenced your choice of treatment.

a. My self-image b. I want to live as long as possible c. I was concerned about side effects from treatment d. My spouse/partner e. My friends f.. My family g. My religion h. Cost of treatment/my insurance

i. Length of therapy j. My doctor’s recommendation

1

4. **Are there any other important reasons in choosing your breast cancer treatment we that did not cover?**

**For your treatment:** (Choose “1= Strongly Disagree”, “2=Disagree”, “3=Unsure”, “4=Agree”, 5= “Strongly Agree”)

15. It was the right decision: 1 2 3 4 5

16. I regret the choice that was made: 1 2 3 4 5

17. I would choose the same choice if I had to do it over again: 1 2 3 4 5

18. My choice did me a lot of harm : 1 2 3 4 5

19. My decision was a wise one: 1 2 3 4 5

***The questions below are about treatment with radiation.***

20. Was radiation recommended to you? No Yes

21. Did you take radiation? (if no, please skip to question 24) No Yes

22. If you took radiation, did/do you have any long term (longer than 3 months) side effects? No Yes

23. If you had to do it all over again, would you choose radiation? No Yes

***The questions below are about treatment with chemotherapy.***

24. Was chemotherapy recommended to you? No Yes

25. Did you take chemotherapy? (if no, please skip to question 29) No Yes

26. Did you complete your chemotherapy regimen? No Yes

27. If you had to do it all over again, would you choose chemotherapy? No Yes

28. Did/do you have any long term (longer than 6 months) side effects from chemotherapy? No Yes

***The questions below are about treatment with hormone therapy***

29. Were anti-hormone pills (e.g. tamoxifen or an aromatase inhibitor) recommended to you? No Yes

30. Did you take anti-hormone pills? (if no, please skip to question 33) No Yes

31. Did you have noticeable side-effects from the anti-hormone pills? No Yes

32. Did you stop taking the pills because of side-effects? No Yes

***Please tell us about your support system for breast cancer***

The following questions relate to the emotional support you received when diagnosed with breast cancer or continue to receive and whether you found them helpful.

33. Did you belong to a support group? No Yes

34. Do you still belong to a group? (if no, please skip to question 36) No Yes

**For the following questions**, please rate from “1= not at all” to “5= extremely,” N/A = not applicable

35. Did you find being in a **support group** helpful? 1 2 3 4 5

2

36. If you **never** belonged to a support group, do you think it might be helpful? 1 2 3 4 5

In battling with breast cancer, women find strength from different areas in their lives. We would like to know:

37. How helpful did you find emotional support from **your family** to you? 1 2 3 4 5

38. How helpful is emotional support from **your friend(s)**? 1 2 3 4 5

39. How about emotional support from **your religion(s)**? 1 2 3 4 5

40. How helpful is emotional support from **your doctor(s)**? 1 2 3 4 5

41. Did you get support from **other healthcare professionals (e.g. social worker, psychiatrist)**? Yes No

42. If “Yes” to Question 41, how helpful did you find **professional support** ? 1 2 3 4 5

**Questions about your general health:**

43. How would you rate your overall health during the past week?

(very poor) 1 2 3 4 5 6 7 (excellent)

44. How would you rate your overall quality of life during the past week?

(very poor) 1 2 3 4 5 6 7 (excellent)

45. Please answer using the following selection: 1=not at all; 2=a little; 3=quite a bit; 4=very much

a. Do you have any trouble doing strenuous activities, like carrying a heavy shopping bag or a suitcase?

1 2 3 4

b. Do you have any trouble taking a long walk? 1 2 3 4 c. Do you have any trouble taking a short walk outside of the house? 1 2 3 4 d. Do you need to stay in bed or a chair during the day? 1 2 3 4 e. Do you need help with eating, dressing, washing yourself or using the toilet? 1 2 3 4

46. In the  **past seven days**, have you been distressed or bothered by: (Choose “1= not at all” to “5= extremely”)

a. Faintness 1 2 3 4 5 b. No interest in daily activities 1 2 3 4 5 c. Nervousness 1 2 3 4 5 d. Chest pain 1 2 3 4 5 e. Feeling lonely 1 2 3 4 5 f. Feeling tense 1 2 3 4 5 g. Feeling nauseous/want to vomit 1 2 3 4 5 h. Feeling blue 1 2 3 4 5 i. Feeling scared 1 2 3 4 5 j. Shortness of breath 1 2 3 4 5 k. Feeling worthless 1 2 3 4 5 l. Have had panic episodes 1 2 3 4 5 m. Numbness or tingling 1 2 3 4 5 n. Feeling hopeless 1 2 3 4 5

o. Feeling restless 1 2 3 4 5

3

p. Body weakness 1 2 3 4 5 q. Thought of hurting yourself 1 2 3 4 5 r. Have had uncontrollable fear 1 2 3 4 5

47. In the  **past seven days**, have you experienced these problems? (1=not at all; 2=a little; 3=quite a bit;

4=very much)

a. Did you have a dry mouth? 1 2 3 4 b. Did food and drink taste different than usual? 1 2 3 4 c. Were your eyes painful, irritated or watery? 1 2 3 4 d. Have you lost any hair? 1 2 3 4 e. Were you upset by the loss of your hair (if applicable)? 1 2 3 4 f. Did you feel ill or unwell? 1 2 3 4 g. Did you have hot flushes? 1 2 3 4 h. Did you have headaches? 1 2 3 4 i. Have you felt physically less attractive as a result of your disease or treatment? 1 2 3 4 j. Have you been feeling less feminine as a result of your disease or treatment? 1 2 3 4 k. Did you find it difficult to look at yourself naked? 1 2 3 4 l. Have you been dissatisfied with your body? 1 2 3 4 m. Were you worried about your health in the future? 1 2 3 4 n. Did you have any pain in your arm or shoulder? 1 2 3 4 o. Did you have a swollen arm or hand? 1 2 3 4 p. Was it difficult to raise your arm or to move it sideways? 1 2 3 4 q. Have you had any pain the area of your affected breast? 1 2 3 4 r. Was the area of your affected breast swollen? 1 2 3 4 s. Was the area of your affected breast oversensitive? 1 2 3 4 t. Have you had skin problems on or in the area of your affected breast (e.g., itchy, dry, flaky)?

1 2 3 4 u. Were you limited in doing either your work or other daily activities? 1 2 3 4 v. Were you limited in pursuing your hobbies or other leisure time activities? 1 2 3 4 w. Did pain interfere with your daily activities? 1 2 3 4

x. Have you had difficulty in concentrating on things, like reading a newspaper or watching television?

1 2 3 4

48. **During the past four weeks**: (1=not at all; 2=a little; 3=quite a bit; 4=very much)

a. To what extent were you interested in sex? 1 2 3 4

b. To what extent were you sexually active (with or without intercourses)? 1 2 3 4

c. If you have been sexually active, to what extent was sex enjoyable for you? 1 2 3 4

4

d. Do you think that your breast cancer or its treatments have interfered with your sex life?

**Please tell us about yourself**

49. What is your age? years old

1 2 3 4

50. What is your race or ethnicity? Please check as many boxes as apply.

White Black Asian Hispanic Other Prefer not to answer

51. What is your relationship status (please check all that apply)?

Single Married Divorced Widowed Partnered/Cohabitating

52. What was the highest level of education you have completed?

Some high school or less High school diploma or GED Associates degree

Bachelor’s Degree Graduate or professional degree

53. How many children do you have? I have: son(s) and daughter(s)

54. Have you gone through menopause? No Yes If YES, at what age? years old

55. Did you have health insurance coverage for your breast cancer treatments?

Yes, completely covered Yes, but I have to pay as well ( % paid by myself) I do not have insurance

56. What types of health insurance do you have? Please write in here:

57. Has your physical condition or medical treatments caused you financial difficulties? Not at all A little Quite a bit Very much

58. Are you currently physically disabled? No Yes

If yes, is it because of breast cancer? No Yes Partially (I have another disability as well)

59. Have you had a recurrence of your breast cancer/has your breast cancer come back (including a different type of breast cancer)? Yes No

60. How much do you worry about if the cancer may come back? 1 2 3 4 5

61. How much do you worry about dying from breast cancer? 1 2 3 4 5

62. **0n the back page, please write down any comments you may have for us.**

End of survey. Please enclose the survey in the self-addressed, stamped envelope and mail it back to us. We have also enclosed a postcard for you to fill out and send back to us. All postcards will be entered into a raffle for prizes.

Please mail the post card separately. Thank you very much!

5
